# Cerebellar Mechanisms Underlying Autism-like Cognitive Deficits in Mouse Offspring with Prenatal Valproic Acid Exposure

**DOI:** 10.3390/toxics13100833

**Published:** 2025-09-30

**Authors:** Juan Wang, Xu-Lan Zhou, Zi-Han Ma, Li Liu, Qian Zhou, Jia-Wei Wen, Jia-Hui Wen, Hui Su, Yu-Han Zhang, Xiao-Chun Xia

**Affiliations:** 1 Department of Public Health and Medical Technology, Xiamen Medical College, Xiamen 361023, China; wang112976juan@xmmc.edu.cn (J.W.); zxl@xmmc.edu.cn (X.-L.Z.); zihan_ma0317@163.com (Z.-H.M.); 18950184521@163.com (Q.Z.); 19959568612@163.com (J.-W.W.); jiahui_wen07@163.com (J.-H.W.); zhangyuhan1@xmmc.edu.cn (Y.-H.Z.); 2 School of Public Health, Fujian Medical University, Fuzhou 350122, China; 990712@fjmu.edu.cn (L.L.); suhui625@163.com (H.S.)

**Keywords:** autism spectrum disorder (ASD), cerebellum, valproic acid (VPA), data-independent acquisition (DIA), proteomics, cognition, synaptic neurotransmission, axon guidance

## Abstract

Autism spectrum disorder (ASD) is a complex neurodevelopmental condition characterized by impairments in social communication and repetitive behaviors, involving various brain regions. Emerging evidence highlights the critical role of the cerebellum in the pathophysiology of autism; however, the underlying molecular mechanisms remain poorly understood. This study aimed to establish a prenatal valproic acid (VPA)-induced mouse model of ASD and explore the potential molecular mechanisms underlying cerebellar ASD-like phenotypes through DIA-based proteomics and bioinformatics analyses. Significant cognitive impairment and anxiety-like behaviors were detected using an open field test and novel object test following VPA exposure, respectively. Additionally, reduced numbers of Purkinje cells with irregular arrangement were observed in the cerebellum. Furthermore, cerebellar proteomics analyses revealed that they identified 193 differentially expressed proteins (DEPs) involved in multiple pathways, including axon guidance, glutamatergic synapse, long-term potentiation, and calcium signaling, among others. Notably, dysfunction of glutamate receptor signaling and disruptions in axon-guidance signaling appear to be major molecular mechanisms underlying cerebellar impairment. Together, these findings suggest that Grin2b may serve as a critical molecule linking synaptic neurotransmission and neurodevelopmental disorders. Thus, Grin2b may represent a potential therapeutic target for addressing cognitive impairment in ASD.

## 1. Introduction

Autism spectrum disorder (ASD) encompasses several conditions characterized by common core symptoms, including social interaction impairments, cognitive deficits, and repetitive behaviors. Although the prevalence of ASD varies across countries and regions, its global incidence has shown a consistent upward trend [1,2,3,4]. The latest epidemiological data reported that the prevalence of ASD in the United States has reached 32.2 per 1000 children, equivalent to 1 in 31 children [5]. Importantly, ASD accounts for 11.5 million disability-adjusted life-years (DALYs) across all age groups, with an age-standardized DALY rate of 147.6 per 100,000 people [6]. These findings indicate that ASD represents a significant public health challenge. Consequently, it is essential to conduct etiological research, identify early diagnostic markers, and develop new therapeutic targets for ASD intervention.

Studies on the etiology of ASD have predominantly focused on abnormal hippocampal function [7,8,9,10]. The pathogenesis of ASD is associated with neuronal damage, synaptic function impairment, neuroinflammation, and other related factors [11,12,13,14,15]. The cerebellum, a brain region primarily responsible for motor learning and coordination, has been strongly implicated in ASD [16,17,18,19]. Increasing recognition has been given to the cerebellar involvement in a range of non-motor functions, including cognitive processes and social behaviors [20,21,22]. Regarding the cerebellar vermis, studies have demonstrated that the volumes of vermian lobules VI and VII in autistic patients are significantly smaller compared to those in healthy controls [23]. Additionally, Clausi et al. reported social cognitive deficits in individuals with cerebellar degeneration [24]. Postmortem studies have consistently identified cerebellar pathology as one of the most reliable findings in ASD patients [25]. Notably, cerebellar injury during the early postnatal period has been linked to an elevated risk of ASD [26]. Collectively, these findings underscore the involvement of cerebellar abnormalities in the pathophysiology of ASD. Nevertheless, the molecular mechanisms underlying cerebellar contribution to ASD, particularly in the context of social cognition, remain to be fully elucidated.

Quantitative proteomics aims to accurately and precisely quantify protein abundance changes while elucidating the underlying mechanism of cellular function and pathogenesis. Data-independent acquisition (DIA)-based proteomics technology integrates the strengths of traditional proteomics approaches, emerging as a powerful tool for biomarker screening and molecular mechanism exploration [27]. Our prior work utilizing DIA proteomics highlighted the prominent role of the neuroimmune response in ASD [28]. This study aimed to investigate the potential molecular mechanisms underlying cerebellar ASD-like phenotypes through DIA-based proteomics and bioinformatics analyses. A classical valproic acid (VPA)-induced mouse model of ASD was established, with behavioral assessment and morphological evaluations conducted. Subsequently, mass spectrometry was used to identify differentially expressed proteins (DEPs) in the cerebellum between VPA-treated and control groups. We further explored the potential implications of these findings for the pathogenesis of ASD-related cognitive impairment and anxiety-like behaviors. Our results support the hypothesis that the aberrant expression of ASD-associated proteins in the developing cerebellum contributes significantly to the etiology of the disorder.

## 2. Materials and Methods

### 2.1. Animals and Treatment

C57BL/6 mice were purchased from the Experimental Animal Centre of Xiamen University (Xiamen, China). Mice were housed in individually ventilated cages under controlled conditions (20 ± 2 °C, 12 h light/dark cycle) and had free access to food and water. All animal experiments comply with the Animal Research: Reporting of In Vivo Experiments (ARRIVE) guidelines and are carried out in accordance with the National Research Council’s Guide for the Care and Use of Laboratory Animals. The experiments were conducted in accordance with the National Institutes of Health Guide for the Care and Use of Laboratory Animals and were approved by the Xiamen Medical College Animal Ethics Committee (Approval No. 20220910001).

Prenatal VPA exposure in rodents has been established as a reliable experimental model to study the pathophysiology of ASD [29,30]. After 1 week of acclimatization, 8-week-old male and female mice were paired (20 ± 2 g, 1:2) for mating overnight. The vaginal secretion was collected the next morning, and the day on which spermatozoa were detected was designated as gestational day 0.5 (GD 0.5). Pregnant female mice were randomly grouped into control and VPA groups. The VPA group received a single intraperitoneal injection of VPA (600 mg/kg in saline, Sigma, Saint Louis, MO, USA) on GD 12.5, while the control group was injected with an equivalent volume of saline. Male offspring were weaned on postnatal day 28 (PND 28) and grouped by maternal treatment.

### 2.2. Behavioral Tests

**Figure 1:**
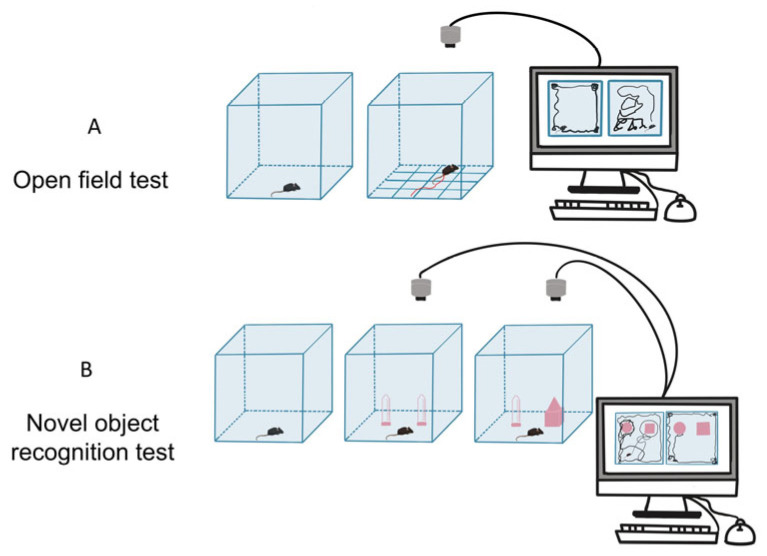
Schematic diagram of autism-like behavioral tests. (**A**) Open field test (OFT). (**B**) Novel object recognition (NOR) test.

Based on the previous research protocols [31,32], the open field test (OFT) was conducted to evaluate psychomotor performance and anxiety-like behaviors in the experimental mice on PND35 (Figure 1A). Male offspring were placed in the central zone of the OFT arena (40 cm × 40 cm × 40 cm, length × width × height), and their behaviors were video recorded for 15 min using a camera mounted above the arena. Each experimental group consisted of 8 male offspring. The total distance traveled and the duration spent in the central zone were analyzed for each offspring using SMART software (Version 3.0.06). The arena was thoroughly cleaned with 75% ethanol between consecutive tests to eliminate residual olfactory cues.

The novel object recognition test (NOR) was performed based on the previous study with minor modification [33] on PND 36-37. Both control and VPA-exposed offspring were placed in an open field arena (a square box, 40 cm× 40 cm × 40 cm) to evaluate the cognitive function based on the innate tendency of offspring to recognize a novel object in the presence of a familiar one in their environment. Each experimental group consisted of 8 male offspring. As shown in Figure 1B, during the first session of the test (habituation phase), mice were presented with two similar objects (cylinders) and allowed to freely explore the open field for 5 min. Following a 24 h delay, in the second session (exploration phase), one of the two cylinders was replaced with a new object (a cube), and the test mouse was then allowed to freely explore the arena for another 5 min. The duration of exploration directed toward the novel object was used to derive indices of recognition memory, including the discrimination index and preference index.

Upon completion of behavioral testing, male offspring were euthanized under ether inhalation anesthesia and subsequently perfused transcardially. Cerebellar tissues were then collected for subsequent histological and proteomic analyses.

### 2.3. Hematoxylin and Eosin (H&E) Staining

H&E staining was conducted to examine histological changes in the cerebellum, following our previous study with modifications [34]. For each experimental group, tissues from 3 male offspring were rinsed with phosphate-buffered saline (PBS) and immediately fixed in 4% paraformaldehyde. Subsequent tissue processing included dehydration, embedding in paraffin (Leica AG, Frankfurt, Germany), sectioning into 5 μm-thick slices, and staining with H&E. Cerebellar Purkinje cells were then observed and analyzed under a light microscope (Olympus Co., Tokyo, Japan).

Purkinje cells were quantitatively analyzed using ImageJ software (Version 1.54p). Purkinje cells were manually counted in each visual field. Two visual fields were observed for each section, with a total of six visual fields observed in each group. Finally, the total numbers of Purkinje cells in each group were statistically analyzed.

### 2.4. DIA-Based Quantitative Proteomics Analysis

To further explore the key role of the cerebellum in the pathogenesis of ASD, DIA-based proteomics analysis was performed according to our previous protocols [28,34].

#### 2.4.1. Protein Extraction and Digestion

For each experimental group, cerebellar tissues from 4 male offspring were collected on the day after the completion of behavioral tests. Briefly, samples were placed in a lysis buffer (containing 7 M urea, 2 M thiourea, and 20 mM tris-HCl, pH 8.4) and subsequently homogenized using an automatic grinder. The supernatant was collected via centrifugation at 25,000× *g* for 15 min at 4 °C. Then, the protein sample was treated with 10 mM DTT and incubated at 37 °C for 30 min. It was incubated with 55 mM iodoacetamide (IAM) in darkness for 45 min at room temperature. Subsequently, five volumes of precooled acetone were added, and the resulting mixture was stored at −20 °C for 2 h. After that, it was centrifuged at 25,000× *g* for 15 min at 4 °C. The dried precipitated proteins were resolved with SDS-free lysis buffer and centrifugated at 25,000× *g* for 15 min at 4 °C. Protein concentration was quantified using the Bradford assay. The protein solution (100 µg) was digested with 2.5 μg MS-grade modified trypsin at 37 °C for 4 h. The digestion reaction was terminated by adding trifluoroacetic acid. Finally, the supernatants were collected and subjected to desalting using a Strata X column. After vacuum-drying, the samples were stored at −20 °C until the next analysis.

#### 2.4.2. DIA Analysis

High-pH reverse-phase separation was conducted using a liquid chromatography system (LC-20AB; Shimadzu, Tokyo, Japan). The elution profiles were monitored at a wavelength of 214 nm, with fractions collected at one-minute intervals. Based on the chromatographic elution peaks, the collected fractions were pooled to yield 10 components, which were subsequently freeze-dried for further analysis. The peptides were reconstituted in 0.1% (*v*/*v*) formic acid and analyzed using a nanoElute liquid chromatography system (Bruker Daltonics, Billerica, MA, USA). Peptide separation was achieved at a constant flow rate of 300 nL/min with the following gradient program: initial linear gradient from 2% to 22% mobile phase B (100% acetonitrile containing 0.1% formic acid) over 45 min, followed by a gradient increase from 25% to 35% mobile phase B over 5 min, a subsequent ramp from 35% to 80% mobile phase B within 5 min, and a final hold at 80% mobile phase B for 5 min.

The separated peptides were ionized via a nano-electrospray ionization (nanoESI) source and subjected to tandem mass spectrometric analysis using a timsTOF Pro system (Bruker Daltonics, Billerica, MA, USA). For DDA (data-dependent acquisition) analysis, the main parameters were set as follows: ion source voltage at 1.6 kV; MS scan range of 100–1700 *m*/*z*; MS/MS cumulative scan time of 100 ms; ion fragmentation mode was CID. Fragment ions were scanned in TOF. Precursor for MS/MS scan satisfied the following criteria: charge range 0 to 5+, top 10 precursors with intensity over 1E4. For DIA (data independent analysis), the main settings were ion source voltage 1.6 kV; the MS scan range was 302–1077 *m*/*z*, which was evenly divided into 32 consecutive windows for MS/MS scanning. Fragment ions were scanned in CID. The fragmentation energy was 10 eV, and the mass width of each window was 25. The cycle time of a DIA scan was 3.3 s.

#### 2.4.3. Data Analysis

The raw DIA data files were analyzed using the iRT peptides for retention time calibration. Data was reviewed based on the UniProtKB/SwissProt *Mus musculus* proteome database (last accessed on 9 May 2024). Search parameters were set as follows: enzyme was trypsin; missed cleavage sites allowed was 2; fixed modification was with carbamidomethyl (C); variable modifications with oxidation (M), acetyl (protein N-term), Gln->pyro-Glu (N-term Q), and deamidated (NQ); fragment mass tolerance of 0.25 Da; peptide mass tolerance of 50 ppm; and MS-MS ion tolerance of 0.1 Da. Spectronaut^TM^ v17.0 (Biognosys, Zurich, Switzerland) was used to construct a spectral library. A spectral library was predicted through the deep learning algorithm in SWATH-MS. The R package MSstats version 4.0 (V4.0) was used to screen differentially expressed proteins (DEPs) according to the absolute log2FC ≥ 1.0 with a *Q*-value of <0.05. The final results were filtered at the precursor ion and protein levels with a 1% false discovery rate (FDR).

Subsequently, relevant databases were used for bioinformatic analysis of DEPs. The UniProt database was used to annotate the GO of the proteome, and DEPs were classified based on three categories of GO annotations, namely biological processes (BP), cellular components (CC), and molecular functions (MF). The KEGG database was used for pathway enrichment analysis. Generally, the functions with *p*-value ≤ 0.05 were regarded as significantly enriched. The Metascape database (https://metascape.org, accessed on 10 December 2024) also uses the Kappa similarity algorithm to absorb most redundancies of biological terms into representative clusters. Heatmap was plotted by https://www.bioinformatics.com.cn (last accessed on 10 December 2024), an online platform for data analysis and visualization [35]. To deeply explore potential protein interactions, protein–protein interaction (PPI) network analysis was conducted using the STRING database (https://cn.string-db.org/, accessed on 10 December 2024). Visualization and interpretation of the biomolecular interaction networks were achieved using Cytoscape software (version 3.9.1).

### 2.5. Western Blotting Analysis

To explore the potential targets for ASD, we analyzed the protein expression of Grin2b, a key protein identified in cerebellar proteomics. Each experimental group consisted of 4 male offspring. Total protein was extracted from cerebellar tissues, and equal amounts of protein were separated by SDS-PAGE, followed by transfer to PVDF membrane using the wet transfer method. After blocking, the membrane was incubated with primary antibodies for Grin2b (1:1000, catalog # 4212T, Cell Signaling Technology, Danvers, MA, USA) at 4 °C overnight. β-actin antibody (1:10,000, catalog # ABL1010, Abbkine, Wuhan, China) was used as a control. Subsequently, peroxidase-conjugated goat anti-rabbit or anti-mouse immunoglobulin G was applied as a secondary antibody (1:10,000, Abcam, Cambridge, UK) and incubated at room temperature for 1 h. After exposure using an ECL reagent, the bands were visualized and imaged using the ECL imaging system (Bio-Rad, Berkeley, CA, USA).

### 2.6. Statistical Analysis

Unpaired *t*-tests were conducted for analyzing behavioral data, histological data, and proteomics data. Data were summarized as means ± SEM. A *p*-value of less than 0.05 was considered statistically significant.

## 3. Results

### 3.1. Prenatal Exposure to VPA Induced Social Cognitive and Anxiety-like Behaviors

**Figure 2:**
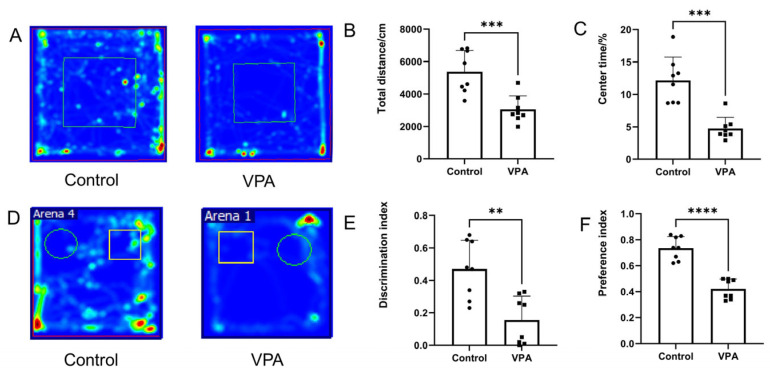
Ethological measurement of mice. (**A**) The trajectories in OFT. (**B**) The total distance traveled in OFT. (**C**) The time spent in the center zone. (**D**) Track of male offspring in NOR test. (**E**) Quantification of the discrimination index and (**F**) the preference index. Discrimination Index = [(Time Spent Exploring Novel Object—Time Spent Exploring Familiar Object)/(Time Spent Exploring Novel Object + Time Spent Exploring Familiar Object)], Preference Index = [Time Spent Exploring Novel Object/(Time Spent Exploring Novel Object + Time Spent Exploring Familiar Object)] × 100%. The results are presented as the mean ± SEM. ** *p* < 0.01, *** *p* < 0.001, **** *p* < 0.0001.

After prenatal VPA exposure in mice, the OFT and NOR tests were performed, respectively. OFT results revealed decreases in total distance traveled and time spent in the central zone, indicating significant anxiety-like behaviors in VPA-exposed offspring (Figure 2A–C). The NOR test demonstrated that both the preference index and discrimination index were significantly reduced in the VPA-exposed offspring (Figure 2D–F), indicating impaired social cognitive function following VPA treatment.

### 3.2. Prenatal Exposure to VPA Induced Significant Cerebellar Defects

**Figure 3:**
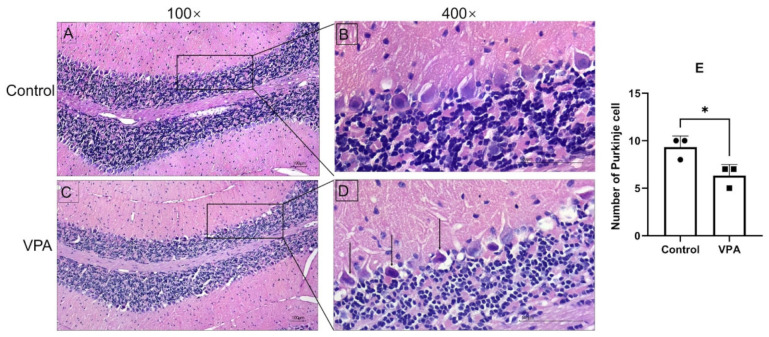
Altered structures of Purkinje cells in the cerebellum of VPA-exposed group. Purkinje cells were arranged neatly and tightly in control group ((**A**), 100×) and ((**B**), 400×), while deeply stained, pyknotic spindle cells were detected in the VPA group ((**C**), 100×) and ((**D**), 400×); black arrows point to pyknotic spindle cells (scale bar, 100 μm). (**E**) The number of Purkinje cells in the cerebellum. The results are presented as the mean ± SEM. * *p* < 0.05.

To investigate the effect of VPA on the cerebellum, H&E staining was performed. The data showed that the cerebellar Purkinje cells were arranged neatly and tightly with clear nucleoli in the control group, while the VPA group showed reduced and irregularly arranged cerebellar Purkinje cells, as well as the presence of deeply stained, pyknotic spindle cells (Figure 3). These results demonstrated that morphological alterations of the cerebellum occurred in VPA-exposed mice offspring.

### 3.3. Quantification of Proteomic Profiling of Prenatal Exposure to VPA and Control

**Figure 4:**
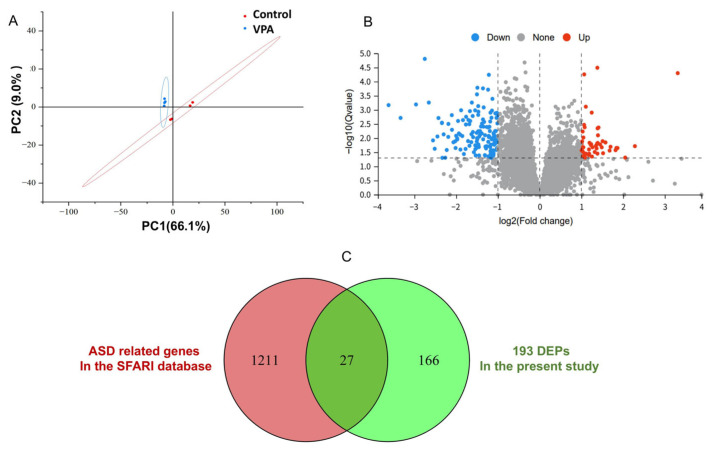
Quantification of differentially expressed proteins (DEPs) between control and VPA-exposed groups. (**A**) Principal component analysis (PCA). The *X*-axis is the first principal component, and the *Y*-axis is the second principal component. (**B**) Volcano plot showing differentially expressed proteins (DEPs) in the cerebellum. The upregulated genes (red) and the downregulated genes (blue) with an absolute log2FC ≥ 1.0 and *Q* < 0.05. (**C**) Venn diagram analysis of DEPs identified in the study and ASD-related genes in the SFARI database.

Quantitative DIA proteomic technology was applied for a proteome comparison between fetal exposure to VPA and control. The MS system stability was supervised by quality control (QC) samples during the whole data-collecting period. A total of 7284 proteins were characterized. Principal component analysis (PCA) results showed that the control and VPA-exposed groups were well separated, and each group of samples displayed well-clustering based on the principal components 1 and 2 (Figure 4A).

A volcano plot was utilized to screen for DEPs. Using a cutoff criterion of absolute log2FC ≥ 1.0 with a *Q*-value (adjusted *p*-value) of <0.05, we identified 193 DEPs in total. Among these, 52 proteins showed increased levels in the VPA group (marked by red dots), while 141 proteins displayed decreased levels (marked by blue dots) (Figure 4B). A comprehensive list of all DEPs is available in the Supplementary Materials (Table S1). When cross-referenced with autism-related genes listed in the SFARI (Simons Foundation Autism Research Initiative) gene database (https://gene.sfari.org/, accessed on 10 December 2024), 27 of these DEPs were found to be included in the SFARI database (Figure 4C). These genes include Grm5, Stx1a, Camk2a, Cdh13, Nrp2, Gpc4, Ntng1, Lin7b, Gabra3, Mast3, Robo2, Brinp3, Syngap1, Clcn4, Sbf1, Tanc2, Grin2b, Dpysl3, Lrrc4c, Elavl2, Cmip, Itpr1, Taf6, Rheb, Dner, Ythdc2, and Nup155. Collectively, these findings strengthen the hypothesis that cerebellar proteomic alterations, particularly involving these ASD-associated genes, may represent a key molecular signature underlying the neurodevelopmental abnormalities observed in the VPA model of ASD.

### 3.4. Functional Enrichment Analysis of DEPs

**Figure 5:**
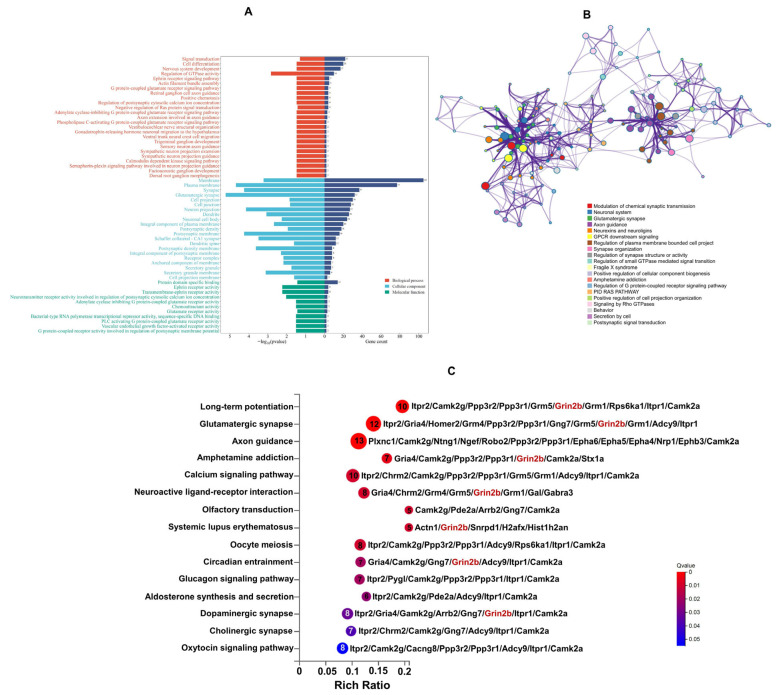
GO enrichment analysis and KEGG pathway analysis of differentially expressed proteins (DEPs). (**A**) GO enrichment analysis. (**B**) Representative sub-network of the interaction. Each node represents the above-enriched terms, colored by cluster ID. (**C**) KEGG pathway analysis. The *X*-axis represents the rich ratio. The number labeled inside each bubble corresponds to the count of DEPs annotated to each pathway, and the color gradient of the bubbles corresponds to the *Q*-value (adjusted *p*-value).

GO functional significance enrichment analysis was conducted to annotate the above-identified DEPs. GO analysis on DEPs obtained 57 significantly enriched GO terms, which included biological process, cellular component, and molecular function. The biological process category comprised 25 distinct processes, encompassing signal transduction, cell differentiation, nervous system development, and regulation of GTPase activity, among others. The cellular component category included 21 types of components, such as plasma membrane, synapse, glutamatergic synapse, and cell junction. The molecular function category consisted of 11 types of functions, including protein domain-specific binding, ephrin receptor activity, transmembrane-ephrin receptor activity, and neurotransmitter receptor activity involved in the regulation of postsynaptic cytosolic calcium ion concentration (Figure 5A).

Furthermore, GO term analysis was performed using the Metascape database to characterize the biological functions of DEPs (Figure 5B). These DEPs were closely associated with several key biological processes, including modulation of chemical synaptic transmission, neuronal system, glutamatergic synapse, axon guidance, neurexins and neuroligins, synapse organization, regulation of synapse structure or activity, and post-synaptic signal transduction. Collectively, these functional annotations suggest that the dysregulated proteins identified in the present research are primarily implicated in synaptic development and signaling pathways.

The KEGG enrichment pathways of the above DEPs were obtained via the KEGG database, and the criterion for significant enrichment was *Q*-value < 0.05. A total of 15 pathways were significantly enriched (Figure 5C). The axon-guidance pathway was associated with the largest number of proteins, followed by the glutamatergic synapse pathway, long-term potentiation, and calcium signaling pathway. Additionally, two other pathways implicated in synaptic transmission, including dopaminergic synapse and cholinergic synapse, were also significantly enriched. These significantly enriched pathways collectively indicate the involvement of key molecular mechanisms underlying neural connectivity and synaptic function.

### 3.5. Protein–Protein Interaction (PPI) Network Analysis of DEPs

**Figure 6:**
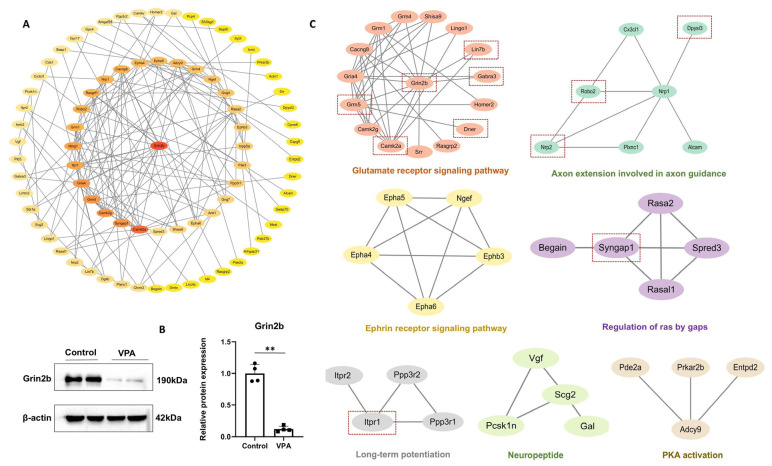
Protein–protein interaction (PPI) network analysis and cluster analysis of DEPs. (**A**) PPI network analysis. Ellipses represent proteins, and gray lines represent interaction partners of DEPs. Each ellipse is color-coded according to score. The larger red and orange ovals show the hub proteins. (**B**) The protein level of Grin2b was detected with Western blotting analysis. ** *p* < 0.01. (**C**) Visualization of cluster analysis. The colors represent the different function clusters. Red dashed boxes represent the proteins identified as ASD-associated genes in the SFARI database.

To better understand the pathogenic mechanisms of ASD in the cerebellum, a PPI network analysis was performed using STRING (last accessed on 17 July 2020) and Cytoscape software. PPI analysis of the DEPs identified the top 29 proteins, including Grin2b, Camk2a, Syngap1, Camk2g, Grm5, Gira4, Itpr1, Ntng1, Grm1, Robo2, Rasgrf1, Nrp1, Cacng8, Epha4, Epha5, Adcy9, Grm4, Ngef, Gng4, Rasa2, Ephb3, Inpp5a, Pak3, Ppp3r1, Gng7, Ank1, Epha6, Shisa9, and Spred3. Noteworthily, Grin2b, a core subunit protein of the glutamate ionotropic receptor NMDA (subunit 2B), was identified as a central node in the PPI network (Figure 6A). Subsequent validation via Western blotting analysis further confirmed the expression level of Grin2b (Figure 6B). As shown in Figure 5C, Grin2b was found to be involved in seven pathways, such as long-term potentiation, glutamatergic synapse, amphetamine addiction, neuroactive ligand–receptor interaction, systemic lupus erythematosus, circadian entrainment, and dopaminergic synapse.

Further PPI cluster analysis of DEPs was conducted by Cytoscape software. The results revealed that the protein network consisted of seven sub-networks (Figure 6C), including glutamate receptor signaling pathway, axon extension involved in axon guidance, ephrin receptor signaling pathway, regulation of Ras by gaps, long-term potentiation, neuropeptide, and PKA activation. Among these sub-networks, the glutamate receptor signaling pathway clustered the largest number of DEPs, with the key members including Grin2b, Camk2a, Camk2g, Grm5, Gria4, Cacng8, Grm1, Grm4, Shisa9, Lingo1, Lin7b, Gabra3, Homer2, Dner, Rasgrp2, and Srr. Notably, Grin2b was identified as the core protein within this pathway, emphasizing its central role in glutamate-mediated signaling processes. Additionally, another sub-network, namely axon extension involved in axon guidance, also clustered seven proteins, including Alcam, Cx3cl1, Dpysl3, Nrp1, Nrp2, Plxnc1, and Robo2. Furthermore, the long-term potentiation sub-network included four key proteins (Itpr1, Itpr2, Ppp3r1, and Ppp3r2), reinforcing the involvement of DEPs in synaptic plasticity mechanisms. Overall, these findings demonstrate that DEPs are functionally enriched in multiple critical neural signaling pathways. Among these, the glutamate receptor signaling pathway appears to play a central role in the cerebellum.

## 4. Discussion

The cerebellum is widely recognized for its role in mediating motor coordination and complex motor behaviors. Meanwhile, growing evidence indicates that perinatal cerebellar damage may be associated with deficits in social and cognitive functions, characteristics frequently observed in ASD [16,36,37]. However, the role of the cerebellum in the pathogenesis of ASD has been poorly studied. In the present study, male offspring exposed to prenatal VPA exposure were found to exhibit significant cognitive dysfunction and anxiety-like behaviors, as assessed by the novel object recognition test and open field test. Furthermore, we also observed a reduction in the number of Purkinje cells in the cerebellum, a finding consistent with previous studies [18,38]. These results suggest a potential relationship between autism-like cognitive impairment and cerebellar defects in mice exposed to VPA during pregnancy.

To investigate the molecular mechanisms, DIA-based proteomic analysis was performed on the cerebellum of a VPA mouse model of ASD. Analysis identified 193 DEPs, including 27 proteins encoded by genes implicated in ASD risk according to the SFARI database. These proteomic alterations, particularly among ASD-associated genes, may represent a component of the molecular pathology underlying neurodevelopmental abnormalities in this model.

Synaptic dysfunction is a key pathogenesis of ASD [39,40]. As a hallmark of many psychiatric disorders, the disrupted synaptic function in ASD can perturb the balance between excitatory and inhibitory neurotransmission, thereby contributing to altered sensory processing, cognitive impairments, and seizure activity. By integrating GO enrichment analysis, KEGG pathway lysis, and PPI network analysis, our findings revealed that pathways closely linked to synaptic function, including synaptic development, synaptic transmission, synaptic plasticity, and neuronal connectivity, were significantly enriched among cerebellar differentially expressed proteins induced by VPA exposure. Guerra et al. [18] reported 162 overlapping genes of cerebellar transcriptome data in the VPA mouse model, and the SFARI database was associated with nervous system development, synaptic signaling, and regulation of synaptic plasticity. Additionally, a prior study demonstrated that VPA primarily modulates the expression of glutamatergic synapses and GABAergic synapses in the rat cerebral cortex [41]. Our results, together with previous findings, provide experimental evidence at the protein level for understanding the link between VPA exposure and ASD-related synaptic dysfunction.

Based on these enrichment pathways, we identified Grin2b as a core protein molecule (Figure 6A). It is directly involved in the glutamate receptor signaling pathway (Figure 6C), which plays a fundamental role in synaptic transmission. Glutamate, the predominant excitatory neurotransmitter, contributes critically to neuronal development and cognitive function through its receptors. Glutamate receptors are categorized as either ionotropic glutamate receptors (iGluRs) or metabotropic glutamate receptors (mGluRs) [42]. iGluRs are non-selective ion channels induced by glutamate that facilitate synaptic transmissions and include N-methyl-d-aspartate (NMDA), 2-amino-3-propionate (AMPA), kainate, and delta receptors. Our findings revealed that a total of 16 DEPs were clustered in the glutamate receptor signaling pathway. Among these, six (Grin2b, Camk2a, Grm5, Lin7b, Gabra3, and Dner) are associated with ASD according to the SFARI database, suggesting a potential link between the glutamate receptor signaling pathway and ASD pathogenesis. As an NDMA receptor subunit (NDMARs), Grin2b is essential for excitatory neurotransmission and has been proposed to contribute to cognitive impairment through the regulation of synaptic plasticity [43,44,45]. Consistent with this, prior studies have linked Grin2b to an increased risk of ASD [46], and Grin2b knockout (KO) models in fish exhibit significantly reduced social preference [47]. Our analysis further supports Grin2b as an ASD-associated gene. Together, these findings suggest Grin2b may serve as a critical molecule linking synaptic neurotransmission and neurodevelopmental disorders. This work provides mechanistic insights into the molecular mechanisms underlying cerebellar synaptic dysfunction in ASD, indicating that Grin2b could represent a potential therapeutic target for cognitive impairment.

In addition to Grin2b, another protein closely associated with NMDA receptors, namely Srr, exhibited significant downregulation in cerebellar proteomic analyses. Srr converts serine into d-serine, a non-natural enantiomer that acts as a co-agonist of NMDA receptors. By enhancing the receptor’s sensitivity to glutamate, d-serine modulates neuronal excitability and synaptic plasticity [48]. The concurrent downregulation of both Grin2b and Srr in the cerebellum of the VPA mouse model further supports the involvement of NMDA receptor dysfunction in impaired synaptic neurotransmission. Furthermore, our analysis identified glutamate ionotropic receptor AMPA type subunit 4 (Gria4) as a key player in the glutamate receptor signaling pathway, which is essential for excitatory synaptic neurotransmission [49]. Pathogenic de novo variants of Gria4 have been linked to intellectual disability and social behavior deficits [50]. We also observed pronounced downregulation of Cacng8, a protein critical for the trafficking and gating of AMPARs [51]. These findings indicate dysregulation of AMPA receptor-mediated synaptic function. Collectively, our results demonstrate coordinated disruptions in both NMDA and AMPA receptor signaling pathways in the cerebellum of VPA-exposed mice, reinforcing the conclusion that glutamate receptor dysfunction contributes to impaired synaptic neurotransmission in this model.

Within the glutamate receptor signaling pathway, Camk2a and Camk2g, two members of the calcium/calmodulin-dependent protein kinase 2 (Camk2) family, exhibit the strongest co-expression patterns with Grin2b. Camk2a, a key regulator of synaptic plasticity, has been shown to have a positive correlation with cognitive ability [52]. Meanwhile, loss of Camk2g function has been implicated in synaptic deficits and cognitive impairment [53]. The concurrent downregulation of both Camk2a and Camk2g may therefore contribute to cognitive deficits associated with cerebellar dysfunction. Although a prior study has reported a relatively minor role for Camk2g in hippocampus-dependent learning and synaptic plasticity [54], this discrepancy may reflect regional specificity within the brain. Camk2g may play a subordinate role in canonical hippocampal synaptic plasticity, while its function could be amplified in the cerebellum critical for cognition.

Notably, our analysis identified that three metabotropic glutamate receptors (Grm1, Grm4, and Grm5) involved in the glutamate receptor signaling pathway were dysregulated. Emerging evidence links Grm5 dysfunction to ASD pathogenesis [55,56]. Consistently, Grm5 is categorized as an ASD risk gene in the SFARI database. Loss of Grm5 induces ASD-like phenotypes, including impaired social interaction, repetitive behaviors, and anxiety-like behaviors [57]. In the present study, the significant downregulation of Grm5 (Log_2_FC = −2.9638, *p* = 0.0006) suggests that Grm5 may play a critical role in autism-like anxiety behaviors by disrupting cerebellar glutamate transmission. Previous studies reported that increased Grm1 immunoreactivity was associated with ASD-behavioral phenotypes in the VPA rat model, including social deficit, repetitive conduct, and anxious behaviors [58]. Our proteomic analysis further indicates that upregulated Grm1 expression correlates with cognitive deficits and anxiety-like behaviors. Additionally, our findings revealed that significant downregulation of Homer2, a key modulator of Grm1 and Grm5, collectively suggests that glutamate transmission is dysfunctional. Grm4 is reported to contribute to maintaining an appropriate excitatory signaling level by modulating glutamate release [59]. Thus, increased Grm4 expression may disrupt the excitatory–inhibitory balance, resulting in the dysregulation of neurotransmitter systems.

Dysfunction of axon-guidance signaling is thought to contribute to microstructural abnormalities in autism [60]. From the PPI sub-network analysis, seven DEPs were clustered in axon extension involved in the axon-guidance pathway, and all seven proteins exhibited significant downregulation. Notably, three of these proteins (Robo2, Nrp2, and Dpysl3) were identified as ASD-associated genes in the SFARI database. Consistent with our findings, prior studies have reported that Robo2 expression is significantly reduced in the autistic group [60,61]. Two major isoforms of neuropilin (Nrp), Nrp1 and Nrp2, are widely expressed in the developing nervous system and are critical for precise axon pathfinding. Their downregulation could weaken the responsiveness of axons to both attractive and repulsive cues, impairing the formation of precise synaptic connections. Dpysl3, another downregulated ASD-associated protein, plays a pivotal role in axon guidance by regulating neuronal growth cone collapse and facilitating cell migration. Together, the coordinated downregulation of Nrp1, Nrp2, Robo2, and Dpysl3 suggests potential impairment in axon-guidance signaling cascades in autism. Additionally, Eph receptor and ephrin signaling play an important role in axon guidance [62] and synapse formation in developing neurons [63]. Herein, the ephrin receptor signaling pathway was clustered in the present study. These findings suggest that dysfunction in axon-guidance signaling may be a key molecular mechanism underlying cerebellar impairment in the VPA model of ASD.

Although our findings provide valuable insights into the etiology of ASD and offer several advantages, we recognize certain limitations. Given the significantly higher prevalence of ASD in boys compared to girls (approximately 4:1), the present study focused exclusively on male offspring for analysis. We have noted that female offspring in ASD models exhibit increased social deficits, repetitive behaviors, and anxiety-like behaviors [64,65,66]. Therefore, it is essential to further investigate ASD-related traits in female offspring and to explore potential sex differences. Additionally, considering the complexity of protein interaction pathways, the roles of other hub proteins, such as Gria4, Camk2a, and Grm5, also need further investigation.

## 5. Conclusions

The study provided novel insights into the molecular mechanisms underlying cerebellar synaptic dysfunction in ASD following prenatal VPA exposure in male offspring. Cerebellar proteomic alterations were identified as a central molecular feature contributing to neurodevelopmental abnormalities in the context. Bioinformatics analysis revealed that 193 DEPs were involved in critical pathways, including the axon-guidance pathway, glutamatergic synapse pathway, long-term potentiation, and calcium signaling pathway, suggesting the complexity of the underlying mechanisms. Notably, the dysfunction of glutamate receptor signaling and disruptions in axon-guidance signaling emerge as dominant molecular mechanisms contributing to cerebellar impairment. Together, these findings suggest that Grin2b may act as a critical molecule linking synaptic neurotransmission and neurodevelopmental disorders. Consequently, Grin2b represents a potential therapeutic target for addressing cognitive impairment.

## Data Availability

All relevant data of this study are given in the manuscript. Additional data will be provided upon request.

## Supplementary Materials

The following supporting information can be downloaded at: https://www.mdpi.com/article/10.3390/toxics13100833/s1, Table S1: DEPs identified between VPA-treated and control groups.

## Abbreviations

The following abbreviations are used in this manuscript:

ASD	Autism spectrum disorder
DEPs	Differentially expressed proteins
VPA	Valproic acid
DIA	Data-independent acquisition
DDA	Data-dependent acquisition
GO	Gene ontology
KEGG	Kyoto Encyclopedia of Genes and Genomes
NMDAR	N-methyl-d-aspartate receptor
AMPAR	2-amino-3-propionate receptor
