# Supplementary material for: Cerebellar Mechanisms Underlying Autism-like Cognitive Deficits in Mouse Offspring with Prenatal Valproic Acid Exposure
Source: Toxics. 2025 Sep 30;13(10):833. doi: 10.3390/toxics13100833 (PMC12568045; doi:10.3390/toxics13100833)
Supplement: Supplementary file 1 [file toxics-13-00833-s001.zip › toxics-3836753-supplementary.pdf]

Table S1. DEPs identified between the VPA and the control group.

No.	Accessions	Protein name	Gene name	Log2FC	Q value
1	Q3UHL1	CaM kinase-like vesicle-associated protein	Camkv	-3.6215▼	6.77E-04
2	O54983	Ketimine reductase mu-crystallin	Crym	-3.3333▼	0.0019
3	Q3UVX5	Metabotropic glutamate receptor 5	Grm5	-2.9638▼	6.42E-04
4	Q6PGE7	Sodium-dependent proline transporter	Slc6a7	-2.7531▼	1.56E-05
5	P62965	Cellular retinoic acid-binding protein 1	Crabp1	-2.6566▼	5.49E-04
6	Q99MX7	Transmembrane protein 121B	Tmem121b	-2.5558▼	0.0120
7	P47212	Galanin peptides	Gal	-2.5152▼	0.0237
8	Q8CC35	Synaptopodin	Synpo	-2.4499▼	0.0087
9	Q8BRV5	Uncharacterized protein KIAA1671	Kiaa1671	-2.4252▼	0.0020
10	P50153	Guanine nucleotide-binding protein G(I)/G(S)/G(O) subunit gamma-4	Gng4	-2.3418▼	0.0029
11	Q9Z140	Copine-6	Cpne6	-2.3416▼	0.0028
12	Q9D3I6	Keratin-associated protein 7-1	Krtap7-1	-2.3334▼	0.0497
13	Q8BI08	Protein MAL2	Mal2	-2.3005▼	0.0073
14	Q3V0I2	Proline-rich protein 7	Prr7	-2.2625▼	0.0492
15	Q8BG18	N-terminal EF-hand calcium-binding protein 1	Necab1	-2.2270▼	0.0262
16	O35526	Syntaxin-1A	Stx1a	-2.1902▼	0.0090
17	Q8CHT1	Ephexin-1	Ngef	-2.1870▼	0.0031
18	Q9WUC3	Lymphocyte antigen 6H	Ly6h	-2.1053▼	0.0015
19	P41242	Megakaryocyte-associated tyrosine-protein kinase	Matk	-2.1017▼	0.0051
20	Q9QUG9	RAS guanyl-releasing protein 2	Rasgrp2	-2.0670▼	0.0139
21	Q5DTY9	BTB/POZ domain-containing protein KCTD16	Kctd16	-2.0239▼	0.0129
22	P25785	Metalloproteinase inhibitor 2	Timp2	-2.0151▼	0.0223
23	Q9CZS1	Aldehyde dehydrogenase X, mitochondrial	Aldh1b1	-2.0016▼	0.0083
24	P11798	Calcium/calmodulin-dependent protein kinase type II subunit alpha	Camk2a	-1.9745▼	0.0110
25	Q2M3X8	Phosphatase and actin regulator 1	Phactr1	-1.9766▼	0.0101
26	O89114	DnaJ homolog subfamily B member 5	Dnajb5	-1.9553▼	0.0025
27	Q80U40	RIMS-binding protein 2	Rimbp2	-1.9359▼	0.0068
28	P97333	Neuropilin-1	Nrp1	-1.9060▼	0.0075
29	Q8BG89	Protein ZNF365	Zfp365	-1.8989▼	0.0253
30	P97467	Peptidyl-glycine alpha-amidating monooxygenase	Pam	-1.8824▼	0.0010
31	Q9WTR5	Cadherin-13	Cdh13	-1.8536▼	0.0084
32	O35405	5'-3' exonuclease PLD3	Pld3	-1.8378▼	0.0023
33	O35375	Neuropilin-2	Nrp2	-1.8158▼	0.0364
34	Q9QUG3	Prion-like protein doppel	Prnd	-1.7758▼	0.0241
35	P59648	FXYD domain-containing ion transport regulator 7	Fxyd7	-1.7679▼	0.0033
36	Q80UG5	Septin-9	Septin9	-1.7636▼	0.0018
37	Q8BLR2	Copine-4	Cpne4	-1.7084▼	0.0023
38	Q7TMJ8	Phosphoinositide-3-kinase-interacting protein 1	Pik3ip1	-1.7167▼	0.0127
39	Q03517	Secretogranin-2	Scg2	-1.6861▼	0.0032
40	Q922S4	cGMP-dependent 3',5'-cyclic phosphodiesterase	Pde2a	-1.6825▼	0.0186

41	Q80TL4	PHD finger protein 24	Phf24	-1.6717▼	0.0011
42	Q9CZN4	Protein shisa-9	Shisa9	-1.6647▼	0.0025
43	Q8VHW2	Voltage-dependent calcium channel gamma-8 subunit	Cacng8	-1.6380▼	0.0138
44	Q03137	Ephrin type-A receptor 4	Epha4	-1.6344▼	0.0072
45	Q9R0S3	Matrix metalloproteinase-17	Mmp17	-1.6253▼	5.50E-04
46	Q9ERZ4	Muscarinic acetylcholine receptor M2	Chrm2	-1.6053▼	0.0016
47	P51655	Glypican-4	Gpc4	-1.5999▼	0.0061
48	Q6PE13	Proline-rich transmembrane protein 3	Prrt3	-1.5951▼	0.0092
49	Q9Z268	RasGAP-activating-like protein 1	Rasal1	-1.5765▼	0.0314
50	Q6NS65	Uracil nucleotide/cysteinyl leukotriene receptor	Gpr17	-1.5464▼	0.0010
51	Q8QZV4	Serine/threonine-protein kinase 32C	Stk32c	-1.5423▼	0.0179
52	Q8BZA7	G-protein coupled receptor 26	Gpr26	-1.5414▼	0.0245
53	Q9D1T0	Leucine-rich repeat and immunoglobulin-like domain-containing nogo receptor-interacting protein 1	Lingo1	-1.5325▼	0.0409
54	Q8R4G0	Netrin-G1	Ntng1	-1.5152▼	0.0221
55	O88951	Protein lin-7 homolog B	Lin7b	-1.5073▼	0.0064
56	Q9CY18	Sorting nexin-7	Snx7	-1.5072▼	0.0131
57	P26049	Gamma-aminobutyric acid (GABA) receptor subunit alpha-3	Gabra3	-1.5027▼	2.82E-04
58	Q68EF6	Brain-enriched guanylate kinase-associated protein	Begain	-1.5015▼	0.0100
59	Q9QZX7	Serine racemase	Srr	-1.4938▼	0.0109
60	Q8CIN6	CUGBP Elav-like family member 3	Celf3	-1.4919▼	1.64E-04
61	Q8K4J6	Myocardin-related transcription factor A	Mrtfa	-1.4731▼	0.0057
62	Q3U3Q1	Serine/threonine-protein kinase ULK3	Ulk3	-1.4729▼	0.0015
63	Q8BLS7	Ankyrin repeat domain-containing protein SOWAHA	Sowaha	-1.4722▼	7.34E-04
64	Q9DCT8	Cysteine-rich protein 2	Crip2	-1.4681▼	0.0012
65	Q9R226	KH domain-containing, RNA-binding, signal transduction associated protein 3	Khdrbs3	-1.4670▼	0.0167
66	Q3U214	Microtubule-associated serine/threonine-protein kinase 3	Mast3	-1.4629▼	0.0193
67	Q8JZW5	SH2 domain-containing protein 5	Sh2d5	-1.4571▼	0.0206
68	Q63811	Calcineurin subunit B type 2	Ppp3r2	-1.4524▼	0.0407
69	Q6NS52	Diacylglycerol kinase beta	Dgkb	-1.4488▼	0.0088
70	Q9EPU5	Tumor necrosis factor receptor superfamily member 21	Tnfrsf21	-1.4279▼	0.0057
71	Q91XV3	Brain acid-soluble protein 1	Basp1	-1.4266▼	0.0076
72	Q60629	Ephrin type-A receptor 5	Epha5	-1.4048▼	0.0039
73	P31650	Sodium-and chloride-dependent GABA transporter 3	Slc6a11	-1.3883▼	0.0068
74	P31324	cAMP-dependent protein kinase type II-beta regulatory subunit	Prkar2b	-1.3822▼	0.0031
75	Q6P6N5	Sprouty-related, EVH1 domain-containing protein 3	Spred3	-1.3589▼	1.73E-04
76	Q6A028	Switch-associated protein 70	Swap70	-1.3587▼	0.0022
77	Q7TPD3	Roundabout homolog 2	Robo2	-1.3544▼	0.0110
78	Q499E0	BMP/retinoic acid-inducible neural-specific protein 3	Brinp3	-1.3472▼	0.0039
79	Q8BG92	Clavesin-2	Clvs2	-1.3416▼	8.16E-04
80	Q8CBH5	Major facilitator superfamily domain-containing protein 6	Mfsd6	-1.3308▼	0.0027
81	F6SEU4	Ras/Rap GTPase-activating protein SynGAP	Syngap1	-1.3144▼	0.0399
82	Q91YN0	FERRY endosomal RAB5 effector complex subunit 3	Ferry3	-1.3094▼	0.0078

83	Q61016	Guanine nucleotide-binding protein G(I)/G(S)/G(O) subunit gamma-7	Gng7	-1.3078▼	0.0407
84	Q9QXV0	ProSAAS	Pcsk1n	-1.3002▼	0.0199
85	Q6ZPF3	Rho guanine nucleotide exchange factor TIAM2	Tiam2	-1.2846▼	0.0229
86	P70459	ETS domain-containing transcription factor ERF	Erf	-1.2705▼	0.0192
87	Q61036	Serine/threonine-protein kinase PAK 3	Pak3	-1.2688▼	0.0040
88	P51830	Adenylate cyclase type 9	Adcy9	-1.2361▼	1.92E-04
89	P16460	Argininosuccinate synthase	Ass1	-1.2356▼	0.0042
90	Q8BGA3	Leucine-rich repeat transmembrane neuronal protein 2	Lrrtm2	-1.2323▼	0.0134
91	Q61418	H (+)/Cl (-) exchange transporter 4	Clcn4	-1.2277▼	0.0442
92	Q99P58	Ras-related protein Rab-27B	Rab27b	-1.2228▼	0.0084
93	Q9QZC2	Plexin-C1	Plxnc1	-1.2195▼	0.0091
94	Q9DB07	Intraflagellar transport protein 46 homolog	Ift46	-1.2177▼	5.72E-05
95	Q61137	Astrotactin-1	Astn1	-1.2036▼	0.0090
96	Q6ZPE2	Myotubularin-related protein 5	Sbf1	-1.2037▼	0.0039
97	O55026	Ectonucleoside triphosphate diphosphohydrolase 2	Entpd2	-1.1999▼	0.0196
98	A2A690	Tetratricopeptide repeat, ankyrin repeat, and coiled-coil domain-containing protein 2	Tanc2	-1.1940▼	0.0056
99	Q91YI4	Beta-arrestin-2	Arrb2	-1.1882▼	0.0417
100	Q8BR92	Paralemmin-2	Pakap	-1.1849▼	0.0026
101	Q62421	Endophilin-A3	Sh3gl3	-1.1835▼	0.0205
102	A2ALU4	Protein Shroom2	Shroom2	-1.1792▼	0.0013
103	Q01097	Glutamate ionotropic receptor NMDA type subunit 2B	Grin2b	-1.1787▼	0.0289
104	O54818	Tumor protein D53	Tpd52l1	-1.1726▼	0.0170
105	P61226	Ras-related protein Rap-2b	Rap2b	-1.1725▼	0.0343
106	Q62407	Striated muscle-specific serine/threonine-protein kinase	Speg	-1.1671▼	0.0267
107	O35188	Fractalkine	Cx3cl1	-1.1636▼	0.0494
108	Q61553	Fascin	Fscn1	-1.1551▼	0.0019
109	E9Q7T7	Chondroadherin-like protein	Chadl	-1.1532▼	0.0109
110	Q9QWW1	Homer protein homolog 2	Homer2	-1.1494▼	0.0370
111	P70699	Lysosomal alpha-glucosidase	Gaa	-1.1431▼	0.0011
112	Q8C437	PEX5-related protein	Pex5l	-1.1322▼	4.06E-04
113	P0C7L0	WAS/WASL-interacting protein family member 3	Wipf3	-1.1150▼	0.0268
114	Q71M36	Chondroitin sulfate proteoglycan 5	CsDpg5	-1.1148▼	0.0418
115	Q9CQN6	Transmembrane protein 14C	Tmem14c	-1.1135▼	0.0096
116	Q62413	Ephrin type-A receptor 6	Epha6	-1.1084▼	0.0402
117	Q0VGU4	Neurosecretory protein VGF	Vgf	-1.1071▼	0.0369
118	Q7TMM9	Tubulin beta-2A chain	Tubb2a	-1.1065▼	0.0332
119	Q80TL0	Protein phosphatase 1E	Ppm1e	-1.1043▼	0.0161
120	Q07646	Mesoderm-specific transcript protein	Mest	-1.1038▼	0.0067
121	Q9WV69	Dematin	Dmtn	-1.1003▼	0.0073
122	Q7TPR4	Alpha-actinin-1	Actn1	-1.0990▼	0.0116
123	Q61199	Neurexophilin-2	Nxph2	-1.0889▼	0.0170
124	Q3U1N2	Sterol regulatory element-binding protein 2	Srebf2	-1.0807▼	0.0103

125	Q62188	Dihydropyrimidinase-related protein 3	Dpysl3	-1.0742▼	0.0084
126	Q9JJU8	SH3 domain-binding glutamic acid-rich-like protein 1	Sh3bgrl	-1.0683▼	0.0056
127	Q61599	Rho GDP-dissociation inhibitor 2	Arhgdib	-1.0660▼	0.0129
128	Q6P5E6	ADP-ribosylation factor-binding protein GGA2	Gga2	-1.0618▼	0.0013
129	Q8C031	Leucine-rich repeat-containing protein 4C	Lrrc4c	-1.0549▼	0.0133
130	O35621	Phosphomannomutase 1	Pmm1	-1.0508▼	0.0056
131	Q9QWY8	Arf-GAP with SH3 domain, ANK repeat, and PH domain-containing protein 1	Asap1	-1.0508▼	0.0149
132	Q80TK0	AP2-interacting clathrin-endocytosis protein	Btbd8	-1.0467▼	0.0122
133	Q923T9	Calcium/calmodulin-dependent protein kinase type II subunit gamma	Camk2g	-1.0401▼	0.0260
134	Q60899	ELAV-like neuron-specific RNA-binding protein 2	Elavl2	-1.0347▼	0.0019
135	Q61490	CD166 antigen	Alcam	-1.0246▼	0.0225
136	Q63810	Calcineurin subunit B type 1	Ppp3r1	-1.0245▼	0.0013
137	Q8CIP4	MAP/microtubule affinity-regulating kinase 4	Mark4	-1.0177▼	0.0107
138	P27671	Ras-specific guanine nucleotide-releasing factor 1	Rasgrf1	-1.0138▼	0.0030
139	Q9D486	C-Maf-inducing protein	Cmip	-1.0080▼	0.0016
140	E9PZJ8	Activating signal cointegrator 1 complex subunit 3	Ascc3	-1.0004▼	0.0120
141	Q9WVK8	Cholesterol 24-hydroxylase	Cyp46a1	-1.0000▼	0.0208
142	Q9ET01	Glycogen phosphorylase, liver form	Pygl	3.3084▲	5.00E-05
143	Q99K30	Epidermal growth factor receptor kinase substrate 8-like protein 2	Eps8l2	2.2822▲	0.0191
144	Q9Z329	Inositol 1,4,5-trisphosphate receptor type 2	Itpr2	2.0499▲	0.0487
145	Q9EQK7	Protein-S-isoprenylcysteine O-methyltransferase	Icmt	1.8660▲	0.0223
146	P11881	Inositol 1,4,5-trisphosphate receptor type 1	Itpr1	1.8385▲	0.0223
147	P30681	High-mobility group protein B2	Hmgb2	1.8189▲	0.0266
148	P58269	Zinc finger protein DPF3	Dpf3	1.6990▲	0.0202
149	A6X8Z5	Rho GTPase-activating protein 31	Arhgap31	1.6761▲	0.0274
150	P56392	Cytochrome c oxidase subunit 7A1, mitochondrial	Cox7a1	1.5777▲	0.0142
151	P97772	Metabotropic glutamate receptor 1	Grm1	1.5193▲	0.0341
152	O08581	Potassium channel subfamily K member 1	Kcnk1	1.5084▲	0.0128
153	Q80XD1	Beta-chimaerin	Chn2	1.4876▲	0.0210
154	P27661	Histone H2AX	H2afx	1.4491▲	0.0167
155	P41139	DNA-binding protein inhibitor ID-4	Id4	1.4111▲	0.0042
156	P63054	Calmodulin regulator protein PCP4	Pcp4	1.3973▲	0.0079
157	Q62311	Transcription initiation factor TFIID subunit 6	Taf6	1.3910▲	0.0148
158	Q8K4B0	Metastasis-associated protein MTA1	Mta1	1.3875▲	0.0045
159	Q921J2	GTP-binding protein Rheb	Rheb	1.3825▲	3.22E-05
160	Q8JZM4	Delta and Notch-like epidermal growth factor-related receptor	Dner	1.3783▲	0.0154
161	Q64444	Carbonic anhydrase 4	Car4	1.3783▲	0.0159
162	P18653	Ribosomal protein S6 kinase alpha-1	Rps6ka1	1.3639▲	0.0230
163	P54754	Ephrin type-B receptor 3	Ephb3	1.3474▲	0.0186
164	B2RR83	3'-5' RNA helicase YTHDC2	Ythdc2	1.3849▲	0.0352
165	Q02357	Ankyrin-1	Ank1	1.3191▲	0.0309
166	Q9CYL5	Golgi-associated plant pathogenesis-related protein 1	Glipr2	1.2654▲	0.0209

167	Q7TNC9	Inositol polyphosphate-5-phosphatase A	Inpp5a	1.2537▲	0.0447
168	Q8BHJ9	Pre-mRNA-splicing factor SLU7	Slu7	1.2471▲	0.0012
169	Q9Z2W8	Glutamate receptor ionotropic, AMPA 4	Gria4	1.2351▲	0.0164
170	P98191	Phosphatidate cytidylyltransferase 1	Cds1	1.2241▲	0.0173
171	Q33DR2	All trans-polyprenyl-diphosphate synthase PDSS1	Pdss1	1.1944▲	0.0146
172	Q8R1H0	Homeodomain-only protein	Hopx	1.1860▲	0.0354
173	Q6IQX8	Zinc finger protein 219	Zfp219	1.1810▲	0.0250
174	P46097	Synaptotagmin-2	Syt2	1.1784▲	0.0396
175	P97433	Rho guanine nucleotide exchange factor 28	Arhgef28	1.1061▲	0.0352
176	Q5PRF0	HEAT repeat-containing protein 5A	Heatr5a	1.1058▲	7.71E-04
177	C0HKE1	Histone H2A type 1-B	H2ac4	1.0933▲	0.0376
178	Q3UX61	N-alpha-acetyltransferase 11	Naa11	1.0850▲	0.0486
179	P05784	Keratin, type I cytoskeletal 18	Krt18	1.0782▲	0.0041
180	Q91XA2	Golgi membrane protein 1	Golm1	1.0723▲	0.0393
181	P58069	Ras GTPase-activating protein 2	Rasa2	1.0677▲	5.54E-05
182	Q9QZH6	Evolutionarily conserved signaling intermediate in Toll pathway, mitochondrial	Ecsit	1.0649▲	0.0034
183	Q99P88	Nuclear pore complex protein Nup155	Nup155	1.0481▲	0.0095
184	Q9D1G3	Protein-cysteine N-palmitoyltransferase HHAT-like protein	Hhatl	1.0476▲	0.0210
185	Q8BUN5	Mothers against decapentaplegic homolog 3	Smad3	1.0365▲	0.0447
186	P59114	mRNA (2'-O-methyladenosine-N(6)-)-methyltransferase	Pcif1	1.0363▲	0.0301
187	P20152	Vimentin	Vim	1.0343▲	0.0209
188	Q9JMG4	Sodium/potassium-transporting ATPase subunit beta-1-interacting protein 4	Nkain4	1.0297▲	0.0058
189	Q6ZPL9	ATP-dependent RNA helicase DDX55	Ddx55	1.0228▲	0.0311
190	Q99JY4	TraB domain-containing protein	Trabd	1.0182▲	0.0129
191	Q6GQU6	Leucine-rich repeat and immunoglobulin-like domain-containing nogo receptor-interacting protein 3	Lingo3	1.0125▲	0.0265
192	P62315	Small nuclear ribonucleoprotein Sm D1	Snrpd1	1.0058▲	0.0160
193	Q68EF4	Metabotropic glutamate receptor 4	Grm4	1.0042▲	0.0272

▼Downregulated; ▲Upregulated.
